# Mixed-methods characterization of the tasks and factors influencing occupational exposure during biosolids land application

**DOI:** 10.1038/s41370-026-00870-x

**Published:** 2026-04-10

**Authors:** Riley J. L. Demo, Carsten Prasse, Keeve E. Nachman, Sara N. Lupolt

**Affiliations:** 1 Department of Environmental Health & Engineering, Johns Hopkins Bloomberg School of Public Health, Baltimore, MD USA; 2 Risk Sciences and Public Policy Institute, Johns Hopkins Bloomberg School of Public Health, Baltimore, MD USA; 3 Johns Hopkins Center for a Livable Future, Johns Hopkins Bloomberg School of Public Health, Baltimore, MD USA; 4 Department of Policy and Management, Johns Hopkins Bloomberg School of Public Health, Baltimore, MD USA

**Keywords:** biosolids, occupational exposure, land application

## Abstract

**Background:**

Pathogenic and chemical contaminants in biosolids may pose risks to workers during land application. Characterizing biosolids applicators’ exposures and risks requires information about the tasks and processes involved in the transportation, application, and use of biosolids, which is critically lacking.

**Objective:**

This mixed-methods study aims to characterize the tasks and processes that influence occupational exposure to biosolids during land application and generate preliminary task-specific exposure factors to refine exposure assessments for contaminants in biosolids.

**Methods:**

We conducted semi-structured, in-depth interviews with biosolids workers who apply, transport, and/or use biosolids in the United States and Canada. Following a framework approach to analysis, we identified the six tasks that constitute biosolids application and developed and administered a biosolids exposure questionnaire to collect preliminary data to derive exposure factors for use in occupational risk assessments.

**Results:**

Workers described frequent and direct contact with biosolids across six land application tasks: hauling, loading, spreading, post-application field work, cleaning, and maintenance. They mentioned instances of ingestion of biosolids, soil, dust, and aerosolized biosolids (spray), inhalation of dust, gases, odors, and aerosolized biosolids (spray), and dermal exposure to biosolids, soil, and dust. Cleaning and maintaining equipment and spreading biosolids were the most commonly reported tasks. Though workers reported spending the least amount of time cleaning and maintaining equipment, these tasks generally resulted in the greatest direct contact with biosolids.

**SIGNIFICANCE:**

Workers’ descriptions of direct contact with biosolids across all exposure routes indicate that land application represents a high-end exposure scenario that warrants further analysis. Our work fills a gap in understanding the behavior and exposure patterns of professional biosolids land applicators and presents preliminary data that can be used for deriving exposure factors for occupational receptors in biosolids risk assessments.

**Impact Statement:**

Exposure to contaminants in biosolids may result in undue health risks, particularly for workers who interact directly with biosolids when applying them to land as a soil amendment. Our mixed-methods exploration of land applicators’ tasks, behaviors, and exposure patterns offers critical information for accurately assessing occupational exposure and identifying potential points of intervention.

## Introduction

Biosolids are the byproduct of the wastewater treatment process, 60% of which are applied to agricultural, commercial, and residential land as fertilizer in the United States [1]. In some cases, chemical and biological contaminants survive wastewater treatment and may become available for exposure among people who have direct or indirect contact with biosolids [2].

**Fig. 1:**
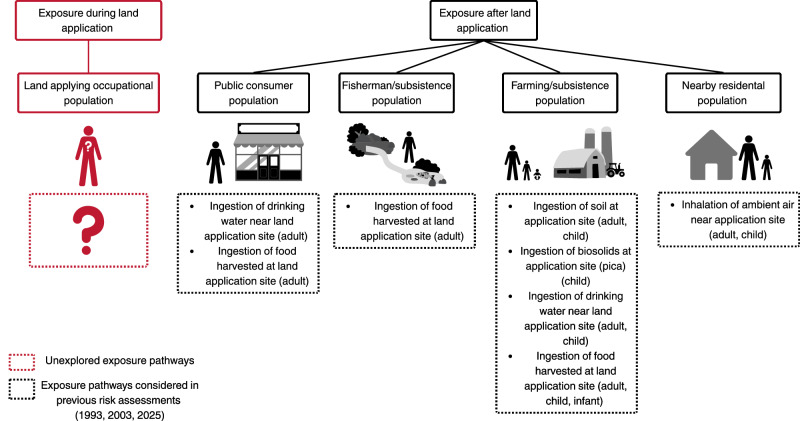
Exposure pathways used in previous risk assessment do not consider land applying occupational populations. Previous risk assessments (1993, 2003, 2025) only consider exposure to biosolids after they are land-applied, missing potential exposure pathways for land-applying occupational populations. The populations (receptors) used in previous assessments are shown in grayscale to the right. The exposure pathways considered in prior assessments are outlined in dotted black boxes. In contrast, this study aims to explore exposure pathways in the previously unassessed occupational population, outlined in red.

The U.S. Environmental Protection Agency (EPA) has conducted several risk assessments to characterize potential health risks arising from exposure to contaminants originating in land-applied biosolids [3–5]. These assessments, the first of which served as the basis for EPA’s Part 503 rule, considered indirect exposures to contaminants in biosolids, i.e., exposures incurred via media that have come into contact with biosolids post-application (Fig. 1). Occupational biosolids exposures, including the direct exposures that occur before and during the application of biosolids to agricultural land, were absent from these risk assessments because the EPA lacked adequate information about worker exposures [3].

Workers who process and land-apply biosolids may have elevated exposures to these materials due to the direct and extended contact required for their jobs [6, 7]. To date, studies of workers that apply biosolids have primarily been in the domain of quantitative microbial risk assessment [8, 9] and have largely relied on general outdoor worker inhalation and ingestion exposure rates from EPA’s Exposure Factors Handbook [10] rather than multi-pathway exposure information specific to tasks involved in occupation. Some work has been done to characterize occupational exposures among workers adjacent to biosolids application, recognizing that agricultural work is variable and involves a number of different tasks that vary in frequency and duration by farm, season, and other factors [11, 12]. One pilot study explored exposures and self-reported health symptoms among wastewater treatment workers [13]. In addition, a combination of qualitative [11, 12] and quantitative [14] studies has provided insights into the tasks involved in agricultural work and the resultant exposures among farmers and other workers on both small and larger farms. Despite this, focus has been absent on workers who transport and apply biosolids to land. Combining qualitative approaches that help scope the universe of job activities with quantitative efforts to enumerate when and how often they occur can be useful in developing the factors needed to estimate exposures incurred by persons engaged in this work. Qualitative approaches that focus on the explanatory factors of exposure (e.g., how exposure occurs or why or how workers do a particular task) are often underutilized in exposure assessment but provide a strong foundation for creating tools to collect quantitative information to inform exposure assessments [15]. In this study, we aim to describe the tasks that comprise the biosolids land application process and explore the contextual factors that may impact workers’ contact with biosolids during land application. We also generate preliminary task-specific data that could be used to derive quantitative exposure factors for use in exposure and risk assessments.

## Methods

### Recruitment

We used purposive sampling to recruit biosolids applicators from across the U.S., prioritizing the 20 states that land-applied the largest amount of biosolids (Table S1) [16]. Eligible workers were ≥ 18 years old and had at least 1 year of experience transporting, applying, and/or using biosolids on agricultural land. To identify workers, we contacted EPA and Occupational Safety and Health Administration (OSHA) staff, asking them to share our study flyer with biosolids workers and managers [17] and/or connect us to state and local departments including staff at the Department of Environmental Quality (DEQ) and Department of Natural Resources (DNR) who, similarly, forwarded our study flyer to workers and managers. Several DEQ and DNR employees also shared lists of contact information for biosolids workers or relevant biosolids companies in their areas. Many states require land application permits that are publicly available on state agency (DEQ/DNR) websites. When available, we used these websites to extract contact information for workers and relevant companies that hauled or applied biosolids. We contacted workers individually by phone or email, reaching out a maximum of three times or until we received a response. We also used snowball sampling, meaning we encouraged study participants to refer their colleagues to our study.

### Data collection

We developed a semi-structured in-depth interview (IDI) guide modeled after similar questions pertaining to activities and soil exposure among agricultural workers (Supplemental Materials) [11]. The guide contained open-ended questions about the land application process, including work tasks, daily, weekly, and seasonal schedules, workplace structure, tools used, and contact with biosolids. We also administered a brief eight-question demographic questionnaire to participants and collected the answers via REDCap, a secure, web-based data collection tool (Supplemental Materials) [18, 19]. Participants gave oral informed consent before each interview began. We audio-recorded all interviews on Zoom and used the automatic transcription feature to record workers’ responses, supplemented with written notes taken by the interviewer. Following the interviews, we used audio recordings to correct any discrepancies or inaccuracies in the automatic transcription, ensuring that the workers’ verbatim responses were accurately reflected. All workers received a $50 e-gift card for their time. We determined saturation of data collection when we heard consistent, recurring descriptions of work experiences across interviews and did not expect to hear new or emergent experiences in conducting future interviews [20].

We developed and administered a Biosolids Exposure Questionnaire (BEQ; Supplemental Materials) to collect time-activity information about the frequency and duration of specific tasks, or meso-activities completed. Briefly, the questionnaire asked workers which of the six tasks (i.e., hauling, loading, spreading, post-application field work, cleaning, and maintenance of equipment) they completed within the last thirty days. For each of the tasks completed, workers were asked to estimate the number of days and the number of hours each day they spent engaged in the task. Workers were also asked about their attire and personal protective equipment worn during each task. Workers also reported subjective indicators of exposure and whether (and how often) they noticed biosolids making contact with specific body parts (e.g., hands, mouth, head). BEQ respondents met similar criteria to those who completed IDI (i.e., were ≥ 18 years old and had at least 1 year of experience transporting, applying, and/or using biosolids on agricultural land) but also had completed the biosolids application process within the past year. The questionnaire was administered via phone, and all data were recorded in REDCap [18, 19]. All respondents received a $25 e-gift card for their time. The Johns Hopkins Institutional Review Board (IRB00028550) reviewed and approved all study tools and protocols.

### Data analysis

We followed an adapted framework approach using five steps [21] to identify key patterns and themes across the interview transcripts, including familiarizing ourselves with the data, developing thematic codes based on our research objective, applying and refining codes to fit the data, organizing and identifying key patterns in the data, and interpreting the findings. For initial familiarization with the data, we thoroughly read the transcripts, noting patterns, similarities, and discrepancies across and within each transcript. We developed thematic a priori, deductive codes using our semi-structured interview guide and added emergent, inductive codes for concepts articulated by the workers that could not be captured using a priori codes (Table S3). For example, we added codes such as “hauling,” “loading,” “spreading,” and “post-application fieldwork” to better articulate the application process, rather than relying on the single a priori code “tasks.” We used ATLAS.ti, a qualitative data management tool, to assist in the organization and application of thematic codes [22]. After coding five transcripts, we organized the codes, noting key themes and patterns that emerged across the transcripts. Throughout this process, RD wrote memos and journal entries shared with the rest of the research team to note emergent themes and to document any potential coder or researcher bias that could have been introduced throughout the analytic process. The research team also met regularly to discuss the memos and insights arising from the analysis. We organized the codes into four groups that explain the themes mentioned by the biosolids workers and factors influencing exposure to biosolids: (1) worker characteristics and industry organization, (2) the biosolids land application process, (3) exposure and contact with biosolids, (4) factors influencing land application tasks and workers’ exposure. These categories provided a framework for our exploration of the data and discussion of the key tasks and explained contextual factors influencing workers’ contact with biosolids during land application. Questionnaire data were summarized using descriptive statistics (i.e., counts, percentages, means, and standard deviations) in Excel.

## Results

### Characteristics of IDI participants

We conducted 11 semi-structured in-depth interviews between May and September 2024. The interviews ranged from 31-71 minutes, with a median of 50 minutes. All participants were male with a median age of 43 years old (range = 26–67 yrs.) (Table 1). All participants had a high school degree with eight continuing their education with some college (*n* = 3), an associate’s degree (*n* = 1), or a bachelor’s degree (*n* = 4) (Table 1). The participants were involved in the transportation, application or use of biosolids across 10 states or provinces in the US and Canada including South Dakota (*n* = 1), Massachusetts (*n* = 1), New Hampshire (*n* = 1), Vermont (*n* = 1), Virginia (*n* = 2), Iowa (*n* = 3), Missouri (*n* = 2), Illinois (*n* = 2), California (*n* = 1), and Vancouver Island (*n* = 2). Three participants reported working in more than one state in the preceding list.

**Table 1:** Demographic characteristics of workers.

Participant	Age (yrs.)	Years working with biosolids (yrs.)	Highest attained education	Participation
Applicator 1	45	22	Some college	Both
Applicator 2	36	5	Some college	IDI only
Applicator 3	29	6	High school completion	Both
Applicator 4	43	5	High school completion	IDI only
Applicator 5	52	2.5	Bachelor’s degree	Both
Applicator 6	42	13	Some college	Both
Applicator 7	26	6	Associate’s degree	Both
Applicator 8	67	5	High school completion	IDI only
Applicator 9	27	12	Bachelor’s degree	BEQ only
Applicator 10	27	5	High school completion	BEQ only
Applicator 11	41	5	Bachelor’s degree	BEQ only
Applicator 12	29	8	High school completion	BEQ only
Applicator 13	55	21	High school completion	BEQ only
Applicator 14	32	6	Some college	BEQ only
Applicator 15	32	22	Bachelor’s degree	BEQ only
Applicator 16	66	30	Associate’s degree	BEQ only
Manager 1	40	24	Bachelor’s degree	IDI only
Manager 2	54	32	Bachelor’s degree	Both
Manager 3	59	33	Bachelor’s degree	IDI only

*IDI* In-depth interview, *BEQ* Biosolids Exposure Questionnaire.

Nearly every participant reported a different job title. Three participants (titles: biosolids supervisor, senior project manager, and agronomy manager) had previous experience in land-applying biosolids but had a management role and were not actively performing land application tasks at the time of the interview. Other job titles included operator (*n* = 2), farm owner, lead operator, farm hand, laborer, driver, and trucking company owner. Each participant had between 2.5 and 33 years of experience working in the biosolids industry (median: 6 yrs.) (Table 1). Those in managerial roles (*n* = 3) had the most experience, with a median of 32 years working with biosolids (range = 24–33 yrs.) while those actively involved in the application process reported a median of 6 years working with biosolids (range = 2.5–13 yrs). Due to the variety in participants’ reported job titles and tasks, we refer to those in managerial positions as “managers” and those actively involved in land application as “applicators.” We refer to all participants generally as “workers.”

### The land application process: workers’ tasks

Workers outlined six tasks involved in the land application of biosolids: (1) hauling biosolids from the wastewater treatment plant (WWTP) to the application site, (2) loading biosolids from the hauling equipment into the spreading equipment, (3) spreading biosolids onto the land, (4) post-application field work at the application site, (5) cleaning biosolids off of equipment or roads and (6) performing maintenance on land application equipment (Table 2). Workers mentioned that land application was typically performed after harvesting crops in the fall (August-November) or before planting crops in the spring (February-May), though the timeline of application varied depending on the geographic location and weather conditions. The workers described different equipment and processes, depending on the type of biosolids they were applying. Before land application, the WWTP often removes excess moisture from biosolids, resulting in a dewatered, “cake” product while other biosolids are left in an unmodified, liquid state [23]. The workers we spoke with mostly applied dewatered biosolids, as they were lighter, easier to transport, and more cost-effective than liquid biosolids. Three workers had experience applying liquid and dewatered products and described the mechanics of the land application for both forms (Table 2).

**Table 2:** Descriptions of land application tasks.

Task	Task definition	Workers’ description
Hauling	Workers picked the biosolids up from the WWTP using a dump truck (dewatered) or an enclosed tanker (liquid). Workers drove the biosolids to the application site where they dumped the biosolids in a loading zone/stockpile (dewatered) or loaded them directly into injection spreaders (liquid).	(Liquid biosolids) *“We take it in semi-trucks with tankers and there is an injector that suctions out the product and inject it into the land.”* – Applicator 2 (Dewatered biosolids) *“It gets dewatered and loaded into bins and then I go there, and I swap out the bins… I pull the loaded one out and put an empty one in and then I take the loaded bin and I drive that to the biosolids application storage facility where I dump it out and then it’s applied immediately or after some period of time.”* – Applicator 1
Loading	Workers took the biosolids from the loading zone/stockpile into the spreading equipment using a pay loader (dewatered) or attached tubes to a hatch on a tanker (liquid) and suctioned the liquid biosolids out of the tanker into the spreader.	(Liquid biosolids) *“Our tanks have vacuum pumps on them, so they’ll create a vacuum and suck the material off of the trailer into the spreader.”* – Manager 1 (Dewatered biosolids) *“The truck unloads the biosolids in a pile on the field… then the big front-end loader picks up the biosolids and loads the biosolids into the spreader and then the spreaders go.”* – Manager 2
Spreading	Workers applied the biosolids on the field using a manure spreader, vertical or horizontal beater (dewatered), or an injection spreader (liquid)	(Liquid biosolids) *“The injector looks like it creates little ditches before the product comes out the back and then once the product comes out the back there’s another pair of disks that bury it back up.”* – Applicator 11 (Dewatered biosolids)*“I get in a tractor that’s hooked to it [the spreader] and pull it behind across the field and engage the PTO [power takeoff] and you got these things that spin on the side, and you open a door and the sludge starts sliding out and it hits them hammers and shoots it out in the field, sprinkles it out. I just drive around in a tractor and punch buttons.”* – Applicator 4
Post-application field work	Workers applied potash, planted crops, harvested, or did other agricultural tasks using large machinery at the biosolids land application site.	*“Once it’s done, I let the farmer know ‘thank you very much that field is done so it’s yours and then if he wants to do more tillage or spread potash he can.”* – Manager 2 *“You could sit in a combine, you know 14, 16 hours a day and just sit there and harvest.”* – Applicator 8
Cleaning	Workers cleaned biosolids from land application equipment, roads, and public spaces using sticks or rakes, hoses, power-washers, or shovels.	*“You got these long scrapers that we use to knock it [biosolids] off the spreader and get in all them spots so we’re not actually grabbing and holding it.”* - Applicator 3 *“We hose it out, use a fire hose or something to basically turn it back to liquid and let it run out.” –* Manager 2
Maintenance	Workers performed routine maintenance (including general upkeep such as greasing equipment, changing oil, or changing cab filters) and breakage maintenance (including fixing or adjusting equipment after a breakage occurs in the field).	*“I show up and if I was going to do applications today, you would go grease your machines, you make sure everything’s good, the oil’s good, the tires are good.”* – Applicator 5 *“If [the spreader chains] bust, you have to go back there an put new links in them to get your spreader right because if one chain breaks the whole spreader won’t work.” –* Applicator 3

The workers hauled dewatered biosolids from the WWTP to the application site using semi-trucks or dump trucks. The dewatered material was then dumped from the semi- or dump trucks into a large pile at the application site, where workers used a payloader to load it into a large box on the back of manure spreaders, horizontal, or vertical beaters. The spreading equipment held the dewatered biosolids in a large box behind an enclosed cab, where the workers operated the equipment. The workers then drove across the application site as the spreaders flung the biosolids out of the back or sides of the box, onto the field.

Workers hauled liquid biosolids using enclosed tankers. At the application site, they attached large tubes to a hatch at the bottom or top of these tankers and siphoned the liquid biosolids into injection spreaders. Once the injection spreaders were loaded, workers sat in a tractor or enclosed cab in front of the spreaders and drove across the field, as the spreader deposited the biosolids beneath the top layer of soil.

After application, workers mentioned several follow-up tasks, including tilling the biosolids into the soil, applying potash as an additional amendment, and planting crops or cover crops at the application site. They noted using similar large-scale agricultural equipment to perform post-application fieldwork, though they did not elaborate further on the specifics of these processes.

All workers, regardless of their other tasks, mentioned that cleaning and maintenance of equipment were regular parts of their job. They described performing daily routine maintenance on hauling, loading, spreading, and post-application equipment at the start of the workday. Occasionally, equipment broke down or malfunctioned, requiring workers to exit their vehicles and perform repairs at the application site. The workers did not specify the tools they used to repair and maintain equipment, but noted that all maintenance tasks were done manually. Cleaning most often occurred following application, before workers moved their equipment to another site. During these periods, some workers mentioned using tools such as rakes, sticks, or scrapers to knock the biosolids off the equipment, while others carried a portable pressure washer to hose the equipment off before leaving. Workers also mentioned cleaning equipment off in the field before performing maintenance and during “off” seasons (winter/summer), when land application was not taking place. In the off-season, they noted using a pressure washer for more thorough cleaning.

Workers performed hauling, loading, spreading, and post-application field work tasks based on the number of colleagues on their team and the training of the team members. In smaller teams (1-3 workers), the workers were responsible for all application tasks, but completed the tasks based on training and necessity:

*“On a typical day it’s just me and one other guy. One person runs a loader, or one person runs the spreader but here recently we’ve had two spreaders going. So, what we do is we both spread then whoever gets back to the loader, will load their spreader and if the other man gets back, he’ll load them too and we just keep alternating.”* – Applicator 3

On larger teams (>3 workers), workers were responsible for only one or two tasks. For example, two workers we spoke with mentioned they had colleagues who were solely responsible for hauling:

*“We have dump truck drivers and that’s all they do is drive the dump truck and all the sludge”* – Applicator 6

*“I’m only on the trucking side so I provide the trucking service for the plant”* - Applicator 2

Another worker mentioned his team had a designated “loading guy”:

*“Our loader guy, he used to work down in the plant where we get our biosolids from and he kind of made his way towards the farm and now he’s just our loader guy”* – Applicator 7

Several workers mentioned additional tasks, outside of biosolids land application, including cleaning biosolids digesters and lagoons, applying animal manure to agricultural land, maintaining compost facilities, and taking care of livestock (cattle). While these tasks are notable in understanding how workers spend their time, they do not directly relate to land application of biosolids and are thus beyond the scope of this work.

### Workers’ exposure to biosolids

Workers described frequent and direct contact with biosolids across all six land application tasks, often involving getting biosolids, soil, and/or dust on their equipment, clothing, and various parts of their body. Several workers described extreme cases where they were completely covered in biosolids:

*“Sadly, I’ve been covered head to toe more times than I like to count.”* – Manager 1

*“I’ll say it’s been more than one time I’ve left work to go home for a shower and come back.”* – Applicator 5

*“I’ve had it in me, on me, all over me.”* – Applicator 1

The workers also described contact inhalation of dust, gases, odors, and aerosolized biosolids (spray) during several land application tasks.

Dust: *“During these summer months it [biosolids] gets very dry and fine, and I empty out the air cleaners of the equipment and there’s a brown dust in there so I’m inhaling all this.”* – Applicator 5

Gases: *“It [biosolids] gives off this hydrochloric acid gas that is very corrosive to the steel of the container so if you don’t get it shoveled out it’ll just eat right through the container and, even with shoveling it out, it still eats through the steel… I don’t use a respirator or anything but just seeing what it does to the insides of the steel containers makes me wonder about my lungs.”* – Applicator 1

Odors: *“You have some with a strong ammonium [sic] smell to it and that’s the ones you got to be most careful about especially this time of year because it’s so hot outside.”* – Applicator 3

Aerosolized biosolids (spray): *“When we clean the equipment, power washers, fire hoses, you know, it kind of sends stuff [biosolids] in all directions”* – Manager 1

**Fig. 2:**
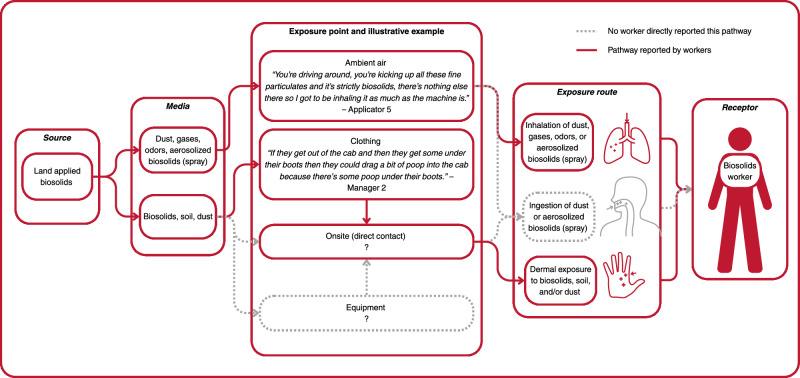
Completed exposure pathways relevant to spreading tasks. This figure depicts complete exposure pathways (source, media, exposure point, exposure route, receptor) described by workers during the spreading task. Spreading was a relatively low-exposure task compared to other tasks, such as cleaning and maintenance. Workers inhaled and ingested dust, gases, odor, and/or aerosolized biosolids (spray) through the ambient air as they drove the spreading equipment across the field. Workers were also exposed dermally to biosolids, soil, and dust that got on their clothing when they got out of their spreading equipment.

Workers reported exposure to biosolids via inhalation and dermal exposure routes across the six land application tasks. When workers spread the biosolids, they described inhaling dust, gases, odors, and/or aerosolized biosolids (spray), and ingestion exposure to dust and aerosolized biosolids (spray) as they drove through the field. They also mentioned direct dermal contact, i.e., getting biosolids, soil, and dust on their clothing and skin after they exited the cab (Fig. 2).

**Fig. 3:**
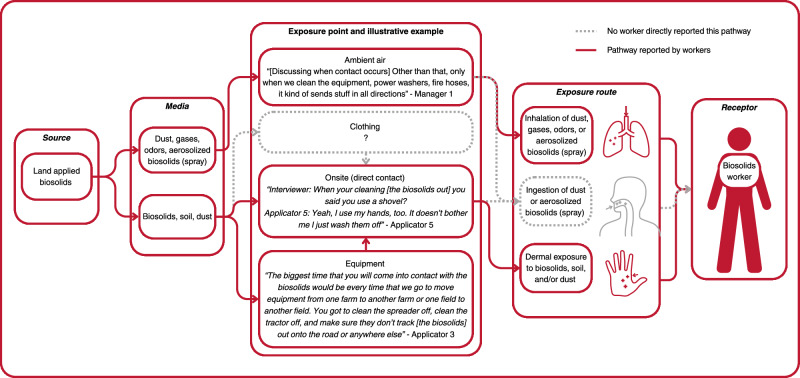
Completed exposure pathways relevant to cleaning tasks. This figure depicts complete exposure pathways (source, media, exposure point, exposure route, receptor) described by workers during the cleaning task. Cleaning was a relatively high-exposure task compared to other land application tasks such as hauling, loading, spreading, and post-application field work. Workers inhaled and ingested dust, gases, odors, and/or aerosolized biosolids (spray) through the ambient air when they hosed off equipment. Workers also directly touched biosolids when cleaning equipmen,t resulting in incidental ingestion and dermal exposure to biosolids, soil, and dust.

All workers consistently reported that cleaning and maintenance resulted in a greater intensity of exposure compared to other tasks. For example, when workers cleaned the equipment, they described dermal contact with biosolids material that was on the equipment and even mentioned touching biosolids directly (Fig. 3). They also mentioned ingesting and inhaling dust and aerosolized biosolids (spray), particularly when using power washers (Fig. 3).

### Factors that impact workers’ exposure to biosolids

#### Workers’ microenvironment

Workers’ exposure to biosolids was largely dependent on whether they were inside or outside the cab spaces of their equipment. Most of the workers’ time was spent operating hauling, loading, spreading, and post-application field work equipment in fully enclosed, air-conditioned cabs elevated above the ground. When inside these cabs, workers noted relatively little direct contact with biosolids:

*“The guys who run the spreading equipment pretty much once they get into their equipment they’re just in their equipment, so they don’t really ever cross paths with the biosolids because they are in their tractors.”* – Manager 2

Workers exited the cab spaces to perform cleaning and maintenance tasks and to transition between hauling, loading, spreading, and post-application field work equipment (i.e., when receiving biosolids from the WWTP, when moving between equipment in the field, when loading liquid biosolids). Outside the cab, workers consistently noted a greater dermal contact with biosolids directly on their head and face, skin and clothing, hands, and inside cuts:

Head and face: *“Every once in a while, [when you’re walking outside the cab] it might fall off the spreader or onto your head or something, so you try to wear a hat.”* – Applicator 6

Skin and clothing: “*When we loop around in the field the biosolids have been spread there so our tires come in contact with the biosolids, and we wash it off so that splashes on your arm sometimes.”* – Applicator 2

Hands: *“It’s on your hands like when the hydraulic fan gets plugged up and you have to fix it.”* – Applicator 5

Inside cuts: *“I’ve slipped and cut my arm and then it’s [the biosolids] come out and got into the cut.”* – Applicator 1

Workers noted inhalation of dust, gases, odors, and aerosolized biosolids (spray) both inside and outside the cab space. They mentioned the intensity was subdued inside the cab, when filters were present, but the odor and some dust were still noticeable:

*“Yeah, the smell will come through the vents so you can still smell it. Sometimes we get special cab filters where it kind of absorbs the smell so it’s not as bad.”* - Applicator 6

When workers got biosolids on their clothes or body outside of the cab, they often carried the biosolids back into the cab space, introducing inhalation of biosolids dust as a new route of exposure:

*“Because you have it [biosolids] on your feet, you get back inside your vehicle and as it dries off on your boots, it kind of goes onto your floor mat and when you have the air conditioning or heater going, it’s blowing up the stuff inside the cab of the truck” –* Applicator 1

A few workers also mentioned tracking biosolids into their personal vehicles, which they used to travel to and from work. Some workers brought a change of clothes to work to avoid tracking biosolids residues home on their clothing. While take-home exposure can be an additional pathway of exposure to biosolids for the workers and those in their households, our participants did not discuss it in detail.

#### The saturation of the biosolids

Workers explained how a higher water content, or saturation of biosolids, typically results in less direct contact with biosolids. Workers reported handling liquid biosolids less often than dewatered biosolids. During liquid land applications, the biosolids remain in enclosed containers (such as tankers, tubes, or injection spreaders), resulting in less contact for the workers. Workers hauled liquid biosolids using sealed tankers that opened at hatches at the top or bottom and loaded the biosolids into the spreading equipment using tubes. While this requires them to exit the cab more frequently to attach the tubing, all the material is kept inside the tanker or tube, resulting in relatively minimal contact.

*“It’s actually easier with less exposure because we’re set up as wastewater people to handle basically sealed containers so we can put our ports and everything into the container and as long as you’re paying attention and don’t overflow the container, it should never get on you.”* – Manager 1

Workers similarly described less contact with liquid biosolids when spreading, as they used an injection spreader that buried the biosolids a few inches beneath the soil. However, two workers described rare instances of equipment malfunction, accidental spillage, or breakages that resulted in ruptured hoses or overflowed liquid tanks that left their bodies covered in biosolids. Though infrequent, the events represent acute, high-end exposure scenarios that should be considered in exposure assessments.

Workers described more occasions of direct contact with biosolids during dewatered land application. For example, two workers described regularly climbing inside the bin of the hauler (where the biosolids are stored) to shovel out remaining material to place in stockpiles:

*“If [the truck driver] is gonna haul something different the next day, he has to get in there and there may be biosolids stuck in the corners and he’ll have to crawl in the trailer and shovel those biosolids out. You got to physically get in there and get them shoveled out so that’s another way they could be exposed to biosolids.”* – Manager 2

Workers noted more inhalation of dust and particulate matter stirred up by the dewatered spreading process, relative to liquid application. They mentioned more contact with their boots and clothes when they got out of the cab, especially if the biosolids were not incorporated or tilled into the soil.

Variability in the moisture content of the dewatered biosolids further modified the nature and timing of workers’ exposure to biosolids. Workers described drier materials as heavy, putting a strain on the equipment, and causing frequent equipment malfunctions that required them to perform maintenance at the application site, outside of the cab space.

*“The drier the material, the more chance you have of the chains breaking because it’s a whole lot heavier material, and it’s making it more stressful on the spreader trying to spread out the biosolids.”* – Applicator 3

On the other hand, excess moisture in the wetter materials made the biosolids “muddy,” causing workers to clean the equipment more often. In both cases, workers were exposed to biosolids outside their equipment. Workers described how drier material resulted in more exposure to dust via inhalation or ingestion, while wetter material stuck to their body, skin, hands, and clothing, resulting in prolonged dermal exposure:

*“When they’re using presses to dewater it, they wouldn’t get as much moisture out so it would come out and be more like mud so then it gets stuck on your body and you kind of just get a paper towel and some hand sanitizer [to wipe it off.]”* – Applicator 1

While liquid application may result in less direct contact than during dewatered application, even within dewatered biosolids application, workers described varying degrees of direct contact with biosolids.

#### Weather

Weather modified the timing of land application tasks and workers’ exposure to biosolids by altering their daily and weekly schedules and the saturation of the biosolids. Workers noted land application occurs according to favorable weather conditions rather than predefined schedules or within distinct seasons. All the workers agreed that land application could not be conducted during snow, ice, or rain events. Some workers applied year-round, only stopping for snow or ice:

*“I apply pretty much year-round. You can’t apply when there’s snow on the ground, obviously, because the snow will act as a transport vehicle for the biosolids. If you put it on top, it will accelerate into streams and rivers, so we don’t apply when there’s snow.”* – Applicator 4

Other workers noted that they only applied in the fall because the springtime was too wet:

*“We know the spreader and tractor weigh over 100,000 lbs., so we don’t want to be on those fields when it’s springtime, when it’s wet, because you get compaction issues. It’s just best to do it in the fall.”* – Applicator 8

Some workers mentioned they did have “typical” working hours (8–10-hour workdays) when the weather was favorable, but even these schedules were flexible and maximized when conditions for application were favorable:

*“Wet periods can be really challenging. If we had a case where we had a site, and it dried out enough to be able to spread, we go spread, and if we don’t finish it that day or it’s close and we have rain coming the next day, they’ll hammer on through and work late just to get it done. Or if it’s a Saturday and we have rain coming on Sunday or Monda,y then they’ll go work on Saturday just to get it done.”* – Manager 3

Workers mentioned that with drier and hotter weather, the biosolids became dry and dusty, resulting in more frequent inhalation of dust and particulate matter:

“*You are around it every day. In the fall, when it’s moist outside there’s not a lot of particulates in the air like there is now but if you’re up there now on a super-hot, dry day the biosolids dry out because they are full of water but as they dry out they become brittle and fine and, like anything else, it breaks down with weather so it can be dusty and it’s very windy up there too so there’s no doubt I inhale quite a bit during the day.”* – Applicator 5

On the other hand, precipitation events such as snow and rain caused the biosolids to become wet and muddy, resulting in more dermal contact via the hands, clothes, skin and body:

*“The farmers don’t like it [when it rains] and neither does my team because we’re dealing with mud at that point, you know, getting the equipment stuck and just making a mess of things in general.”* – Manager 1

As such, weather acted as an upstream factor for exposure, as rain altered the saturation, which modified workers’ exposure. Weather also modified the timing of land application tasks and may be an important consideration when assessing worker exposure.

#### Workers’ actions to reduce their contact

Workers described three intentional behaviors to reduce their contact with biosolids during the land application process. Most workers we spoke with described taking steps to minimize their interaction with biosolids during tasks to avoid contact:

*“We try and keep everybody as far away from actually touching material as we can. There’s nothing wrong with it, it’s perfectly safe it’s just if you don’t have to get dirty, why get dirty?”*- Manager 1

Some workers also described cleaning themselves off before re-entering their cab or their personal vehicles:

*“So then they have boot cleaners on the side of their semi they can wipe their bottom of their boots clean before they get in their semi.”* – Manager 2

Workers also reported wearing personal protective equipment (PPE). For instance, two workers mentioned regularly wearing hats (though it was unclear what type of hat) as sun protection and to avoid getting biosolids on their heads, and one worker mentioned wearing safety goggles. Six workers wore gloves when performing manual tasks outside of the cab, while the other five workers never used them. We noted that workers’ perceived risk of exposure to biosolids may have influenced their use of PPE. Several workers mentioned that they believed biosolids to be “safe” or “not toxic,” drawing comparisons between biosolids and other fertilizers, such as manure:

*“I don’t think it’s [biosolids] as harmful as people think, you know, it’s not toxic or anything like that. It’s just fertilizer.”* – Applicator 4

These perceptions may have played a role in workers’ decisions to wear PPE:

*“It’s on equipment so, I mean, some people wear gloves when there working. I don’t bother; I’m nose blind to it now. I grew up on a dairy farm, so the stigma behind it doesn’t bother me any.”* – Applicator 5

Workers’ actions to reduce their contact fall into three categories in the occupational hierarchy of controls [24], including engineering controls (i.e., staying inside cab space), administrative controls (i.e., cleaning themselves off before re-entering the cab), and PPE use (i.e., wearing hats). While we found that these actions varied, the scope of controls may be helpful in designing interventions to reduce workers’ exposure to biosolids.

### Characteristics of BEQ respondents

We administered the BEQ via telephone to 14 workers between October 2024 and March 2025. All but one participant (93%) was male. Six workers participated in both the IDI and BEQ parts of our study; eight workers participated only in the BEQ portion of the study (Table 1).

All participants had a high school degree, with eight continuing their education with some college (*n* = 3), an associate’s degree (*n* = 2), or a bachelor’s degree (*n* = 5) (Table 1). The workers were in their current role for a mean of 13 years (SD = 12) (range = 2-45) and reported having worked with biosolids for a mean of 14 years (SD = 10) (range = 2.5–32 years).

Thirteen workers reported working with digested biosolids. Nine workers (64%) reported working with class B biosolids. Three workers worked with class A and 1 worked with “Exceptional quality”. Four workers were not sure what type of biosolids they worked with. Most of the workers in this study worked with biosolids with higher water content (e.g., 8 workers (57%) worked with liquid (i.e., < 3% solids) and 9 (64%) worked with cake-like biosolids (15-30% solids).

### Frequency and duration of biosolids contact

Workers reported working “with or in the presence of biosolids or biosolids amended soil” a mean of 10 days (SD = (range = 0–30) and a mean of 5 hours (range = 0–12) on each day they worked within the previous thirty days. On the days they worked, 11 workers spent time outside (mean = 45% of their total worktime), and 13 (93%) workers spent time in a vehicle (mean = 68% of their total workday).

### Frequency and duration of tasks

Of the six tasks (hauling, loading, spreading, post-application field work, cleaning, and maintenance), cleaning was the most common task completed (*n* = 13, 93% of workers), followed by loading, spreading, and maintenance (*n* = 12, 86% of workers). Out of the time engaged in biosolids land-application activities, workers spent the most time spreading (98 hours/month) and hauling (83 hours/month), and the least amount of time doing loading (36 hours/month) and cleaning (19 hours/month) (Table 3).

**Table 3:** Summary of frequency and duration of time spent engaged in six tasks involved in biosolids application.

Task and number of workers completing it	Number of days		Hours/day		Total hours/month	
	Mean (SD)	Range	Mean (SD)	Range	Mean (SD)	Range
Hauling (*n* = 2)	18 (11)	(10-25)	5 (1)	(4-5)	83 (60)	(40-120)
Loading (*n* = 5)	16 (8)	(6-27)	2 (1)	(1-4)	36 (24)	(18-76)
Spreading (*n* = 7)	14 (8)	(5-25)	7 (4)	(2-11)	98 (93)	(18-275)
Post-application field work (*n* = 4)	10 (10)	(2-25)	7 (5)	(2-12)	69 (104)	(10-225)
Cleaning (*n* = 9)	8 (8)	(1-20)	2 (2)	(1-6)	19 (29)	(1-90)
Maintenance (*n* = 7)	6 (9)	(1-26)	3 (3)	(1-10)	40 (97)	(1-260)

### Biosolids contact

Workers observed contact with biosolids varied by task and body part. For example, at least 40% and 25% of workers reported noticing biosolids on their clothing and hands, respectively, during every task (Table S3). Cleaning and maintenance were the tasks with the greatest percentage (of workers (67% and 71%, respectively) noticing biosolids on their hands. Only during cleaning did 3 workers (33%) notice biosolids in their mouths. No workers reported noticing biosolids on their head or face during any task.

Workers reported smelling biosolids during all tasks. Workers reported smelling biosolids at least 70% of the time on average for 5 tasks (i.e., hauling 100%; maintenance = 90%; cleaning = 86%; spreading = 79%; loading = 72%) (Table S4). Workers subjectively ranked their level of biosolids/soil contact for each of the six tasks on a 10-point scale (1 = no contact at all and 10 = high contact). Mean scores ranged from 2 (for spreading and post-application field work) to 7 (for maintenance).

### Attire and personal protective equipment

All workers reported wearing long pants for every task completed (Table S5). Most workers wore boots for all tasks. The percentage of workers reporting the use of protective eyewear ranged from 29% (spreading) to 50% (hauling and post-application field work). Fifty percent of workers reported wearing gloves while hauling; 33% and 29% of workers reported wearing gloves while cleaning and doing maintenance, respectively. None of the workers reported wearing a mask, sneakers, or a face shield for any task.

## Discussion

Understanding biosolids exposures among workers who land apply them is essential for informed decision-making. We used in-depth interviews to define the universe of tasks and activities in the land application process and explore how they may be exposed to biosolids. They reported frequent and direct contact, though their exposures varied across the six land application tasks depending on several contextual factors (i.e., microenvironment, saturation of biosolids, weather, actions taken to reduce contact). Workers’ descriptions of how and under what circumstances they come into contact with biosolids will support the development of robust scenarios that fully encompass the nuances and variability of their exposures.

We further characterized workers’ potential exposure to biosolids during application by generating exposure factors for the six tasks described by workers during the in-depth interviews. We quantified the frequency and duration of each task and found that workers spend the most time spreading and hauling, and the least amount of time loading and cleaning equipment. While occupying the least amount of time, workers reported cleaning and maintaining equipment were the tasks they conducted the most and rated them the most biosolids/soil contact-intensive tasks. We also note variation in the number and workers and the frequency at which they noticed biosolids contact with specific body parts. These data may be informative for developing a task-specific exposure model for estimating occupational exposures to chemicals in biosolids among biosolids applicators.

To our knowledge, our study is the first to draw on the lived experiences of biosolids applicators to qualitatively describe the tasks of land application in their own words. Previous EPA biosolids risk assessments have only assessed exposure and risks among residential populations who likely have a very different exposure experience compared to workers [3]. In contrast, the workers we spoke with described regular, direct contact in the processes of loading and transporting biosolids, and during and immediately after land application, times when non-occupational access is typically restricted. The six tasks described by workers in our study were distinct from other traditional agricultural tasks [11] and did not include work done by WWTP workers. Notably, workers highlighted cleaning and maintenance of land application equipment as two tasks with the greatest potential for direct contact with biosolids; these tasks emerged during interviews and were not included in our research team’s initial conception of the application process. Our findings suggest that workers who land-apply biosolids are uniquely and highly exposed to biosolids, and a distinct land-application scenario should be developed and included in future risk assessments.

Biosolids risk assessments to date have not quantified exposures incurred by workers. In the most recent EPA risk assessment of PFOS and PFOA in biosolids, the authors acknowledged that occupational exposures may be important, but they cited a lack of data about their work schedules, behaviors, the occurrence and concentration of these specific compounds in air, and other factors as limitations that precluded estimation of exposure (they also cited a lack of inhalation and dermal toxicity values as a barrier to estimating risk) [3]. To date, farm families (adults and child residents) are the only receptors with potentially direct biosolids contact for whom exposure has been modeled. In the case of these receptors, the focus on their exposures has been limited to the quantification of incidental ingestion of biosolids-amended farm soils and consumption of crops grown in those soils.

Our interviews made clear that workers routinely ingest, inhale, and have dermal contact with biosolids during the six tasks involved in land application. For this reason, and in recognition of the array of compounds known to occur in biosolids [25], workers involved in land application may be among the most exposed and should be considered in assessments conducted [26] to support risk management. An additional need for quantifying worker exposures and risk is biosolids application-specific exposure factors. In the absence of exposure factors (e.g., incidental soil ingestion rates) specific to these workers, risk assessors typically use general population estimates; however, the US EPA’s confidence in these estimates remains low [26, 27] and their relevance to high soil contact scenarios remains poorly understood [28]. Our previous work has documented that the use of traditional exposure factors for media contact rates and work intensity likely results in underestimation of the true exposures incurred by farmers and other agricultural workers [14]. The results of our interviews make clear that biosolids land application work is unique – even different from other typical farm work – and that task-specific exposure factors may be needed to accurately characterize exposures and risk.

The benefit of our qualitative approach was our ability to identify specific tasks and explanatory factors that modified workers’ exposure. Previous studies have modeled occupational risks of biosolids exposure using gross empirical assumptions of exposure frequency and duration, but these studies do not consider the range of tasks and factors that may impact contact with biosolids [8, 9]. By directly asking workers about the land application process, we were able to explore and describe behaviors and factors that may impact exposure. Many of the modifying factors described in our study align with patterns in agricultural workers’ exposure to soil, described by Lupolt et al., including changes in environmental conditions, tasks, timing of land application, and behavioral/biological characteristics of the workers [12].

In our study, workers also identified the saturation of the biosolids as a factor that affected both the land application process and their exposure. We hope that future studies will collect quantitative data on these factors that can be used to refine exposure assessments. In addition, these factors may be further explored as potential areas of intervention to reduce workers’ exposure. For instance, we found that workers described less intensity of exposure when using liquid biosolids relative to dewatered biosolids, yet the use of dewatered material was more common among our participants. There may be merit in future interventions to focus on exposure during dewatered land application, as our participants indicated this mode of application resulted in greater exposure.

We encountered challenges in identifying and recruiting workers to participate in this study. In some cases, it was difficult to identify persons involved in the land application process. For example, some municipalities have employees who are responsible for land application, but that task may not be their primary job responsibility. As such, it may be difficult to identify the persons engaged in this work from municipality websites or other publicly accessible sources. Similarly, land application may be performed by employees who spend little of their work time behind a desk. As such, reaching them through typical recruitment channels may be difficult because they may not have or monitor an email address or may not have their own dedicated work telephones. Our most successful recruitment strategy was engaging managers and supervisors who had previous experience in applying and were willing to pass along the study information to their colleagues. Further, it is possible that willingness to participate in this study may have been tempered by public attention focused on the issue of contamination of biosolids with per- and polyfluorinated alkyl substances (PFAS) [29, 30]. We believe this may be the reason that our repeated attempts to reach applicators through larger water utilities and companies involved in biosolids handling were unsuccessful.

Given our small sample size, it is possible our data do not represent the full range of workers’ experiences. For example, none of the workers we interviewed described spreading equipment such as splash plates or liquid spray (on top of the soil) that were mentioned in another study [8]. These pieces of equipment may come with additional considerations for exposure that we were not able to explore. The limited number of responses (*n* = 14) to our biosolids exposure questionnaire precluded us from generating robust distributions for use in probabilistic risk assessment and from assessing statistical differences in key exposure factors by task or by type of biosolids applied. Despite the uncertainty arising from our small sample size, we believe these quantitative data are a useful starting point in efforts to begin to quantify applicator exposures to contaminants in biosolids.

Our findings offer a qualitative foundation for understanding exposure factors, or the behaviors and environments that influence land applicators’ contact with biosolids, and an initial attempt to generate data that could be used to inform quantitative exposure factors to model contact with biosolids [10]. This work would be strengthened by further quantitative data collection, particularly data on the frequency, duration, and timing of each task of the identified tasks. A quantitative characterization of the factors our respondents reported as modifying exposure (microenvironment, saturation of biosolids, weather, and actions workers took to reduce their own contact) is also needed; these data should also be incorporated into models to better estimate applicator exposures. These behavioral factors, in combination with field measurements of specific contaminants in biosolids, could form a rigorous foundation for decision-making to protect worker health.

## Supplementary information

Supplementary Material

## Data Availability

The datasets generated and analyzed during the current study are available from the corresponding author upon reasonable request.
