# Supplementary material for: Mixed-methods characterization of the tasks and factors influencing occupational exposure during biosolids land application
Source: J Expo Sci Environ Epidemiol. 2026 Apr 10;36(4):796–806. doi: 10.1038/s41370-026-00870-x (PMC13331729; doi:10.1038/s41370-026-00870-x)
Supplement: Supplementary file 1 — Supplementary Material [file 41370_2026_870_MOESM1_ESM.docx]

**Supplementary Material for “Qualitative characterization of the tasks and factors influencing occupational exposure during biosolids land application”**

Riley J.L. Demo, Carsten Prasse, Keeve E. Nachman, Sara N. Lupolt

**Table of Contents**

**Supplemental tables**

**Table S1.** States targeted for purposive sampling and dry metric done of biosolids land applied per year

**Table S2.** Example of coded interview texts

**Table S3.** Summary of biosolids contact by task and body part

**Table S4.** Summary of subjective biosolids exposure indicators, by task

**Table S5.** Summary of the percentage of wearing select pieces of attire, by task

**Supplemental figures**

**Figure S1.** Completed exposure pathways relevant to hauling tasks

**Figure S2.** Completed exposure pathways relevant to loading tasks

**Figure S3.** Completed exposure pathways relevant to post-application field work tasks

**Figure S4.** Completed exposure pathways relevant to maintenance tasks

**Supplemental information**

In-depth interview guide

Biosolids Exposure Questionnaire

Demographic Questionnaire

**Table S1. States targeted for purposive sampling and dry metric done of biosolids land applied per year**

| State | Dry metric tons of biosolids land applied (ton)* |
| --- | --- |
| Iowa | 345,600 |
| North Carolina | 113,299 |
| Arizona | 79,392 |
| Colorado | 76,853 |
| Oregon | 70,002 |
| Georgia | 65,917 |
| Nebraska | 61,291 |
| Oklahoma | 49,813 |
| California | 44,944 |
| New Hampshire | 44,219 |
| Maryland | 32,139 |
| Pennsylvania | 28,369 |
| Virginia | 21,947 |
| Alabama | 21,443 |
| Idaho | 20,328 |
| Illinois | 20,124 |
| Kansas | 19,758 |
| South Dakota | 19,285 |
| Arkansas | 17,385 |
| *Data from the 2022 National Biosolids Survey | |

**Table S2.** Example of coded interview texts

| Transcript excerpt | Code |
| --- | --- |
| Applicator 3: The biggest time that you will come into contact with the biosolids would be every time that we go to have to move equipment from one, one farm to another farm, or one field to another field is getting back there, and you got to clean, you know, clean the spreader off, clean the tractor off to make sure that they don't track out onto the road or anywhere else. So that's the only time that I really come in contact with it. Besides, we have to, every once in a while, you have to take samples of it to send into the, well, we just take it to the office, and they send it off. For them to study it, look at it. That's the only other time we'll be coming contact with it.    Interviewer: Gotcha. So, when you are like cleaning it off, or like cleaning off the spreader and stuff like that... I know it's silly but could you just like kind of talk about like what you do during those, those cleanings.    Applicator 3: Most of the time we got long scrapers that are 5 or 6 feet long. Most of the time we are not actually like touching them with our hands. You got these scrapers that we use to knock it off the spreader to get in all them spots with so we're not actually like we're not grabbing and holdin’ it. You're just using that to scrape it off the tires scraping off every part of the buggy that normally while you’re spreadin’ is getting coated with it. That's what we're cleaning off so that when we pull out on the road or even from one field to another field to keep it out of the buffer. We use em’ it's only about five- or six-foot bars that we use to clean it off with so we're not actually grabbing it with our hands. We're using them scrapers to scrape it off. | Cleaning, Contact with biosolids, Moving equipment            Contact with biosolids, Taking Samples                  Cleaning equipment, Actions to reduce contact  Cleaning equipment, actions to reduce contact    Cleaning, Cleaning equipment, Moving equipment    Actions to reduce contact, Cleaning Equipment |

**Table S3.** Summary of biosolids contact by task and body part

| Body part | Hauling  (n=2) | Loading  (n=5) | Spreading  (n=7) | Post-application field work (n=4) | Cleaning  (n=9) | Maintenance (n=7) |
| --- | --- | --- | --- | --- | --- | --- |
| **Hands** |  |  |  |  |  |  |
| % of workers noticing contact | 50 | 40 | 29 | 25 | 67 | 71 |
| Mean # of contact events | 10 | 8 | 18 | 5 | 7 | 7 |
| Mean % of time biosolids is in contact | 10 | 8 | 33 | 50 | 57 | 68 |
| **Exposed skin of another body part (not hand)** |  |  |  |  |  |  |
| % of workers noticing contact | 100 | 40 | 29 | 0 | 44 | 57 |
| Mean # of contact events | 7 | 13 | 6 | 0 | 7 | 2 |
| Mean % of time biosolids is in contact | 45 | 6 | 20 | 0 | 33 | 38 |
| **Clothing** |  |  |  |  |  |  |
| % of workers noticing contact | 100 | 60 | 43 | 44 | 78 | 86 |
| Mean # of contact events | 11 | 10 | 12 | 7 | 7 | 2 |
| Mean % of time biosolids is in contact | 90 | 19 | 40 | 33 | 80 | 49 |
| **Mouth** |  |  |  |  |  |  |
| % of workers noticing contact | 0 | 0 | 0 | 0 | 3 | 0 |
| Mean # of contact events | 0 | 0 | 0 | 0 | 1 | 0 |
| Mean % of time biosolids is in contact | 0 | 0 | 0 | 0 | 0 | 0 |
| **Head or face** |  |  |  |  |  |  |
| % of workers noticing contact | 0 | 0 | 0 | 0 | 0 | 0 |
| Mean # of contact events | 0 | 0 | 0 | 0 | 0 | 0 |
| Mean % of time biosolids is in contact | 0 | 0 | 0 | 0 | 0 | 0 |

**Table S4.** Summary of subjective biosolids exposure indicators, by task

| Task and number of workers completing it | Mean % of time smelling biosolids | Mean exposure level  (1-10, 10 – max) |
| --- | --- | --- |
| Hauling (n=2) | 100 | 4 |
| Loading (n=5) | 72 | 3 |
| Spreading (n=7) | 79 | 2 |
| Post-application field work (n=4) | 27 | 2 |
| Cleaning (n=9) | 86 | 6 |
| Maintenance (n=7) | 90 | 7 |

**Table S5.** Summary of the percentage of wearing select pieces of attire, by task

| Task and number of workers completing it | Long pants | Short pants | Long sleeve or jacket | Short sleeve shirt | Boots | Hat | Protective eyewear |
| --- | --- | --- | --- | --- | --- | --- | --- |
| Hauling (n=2) | 100 | 0 | 50 | 100 | 100 | 100 | 50 |
| Loading (n=5) | 100 | 0 | 60 | 60 | 100 | 80 | 40 |
| Spreading (n=7) | 100 | 0 | 57 | 71 | 100 | 86 | 29 |
| Post-application field work (n=4) | 100 | 0 | 75 | 50 | 100 | 75 | 50 |
| Cleaning (n=9) | 100 | 11 | 44 | 66 | 89 | 88 | 33 |
| Maintenance (n=7) | 100 | 0 | 57 | 71 | 100 | 88 | 43 |

**Figure S1.** Completed exposure pathways relevant to hauling tasks


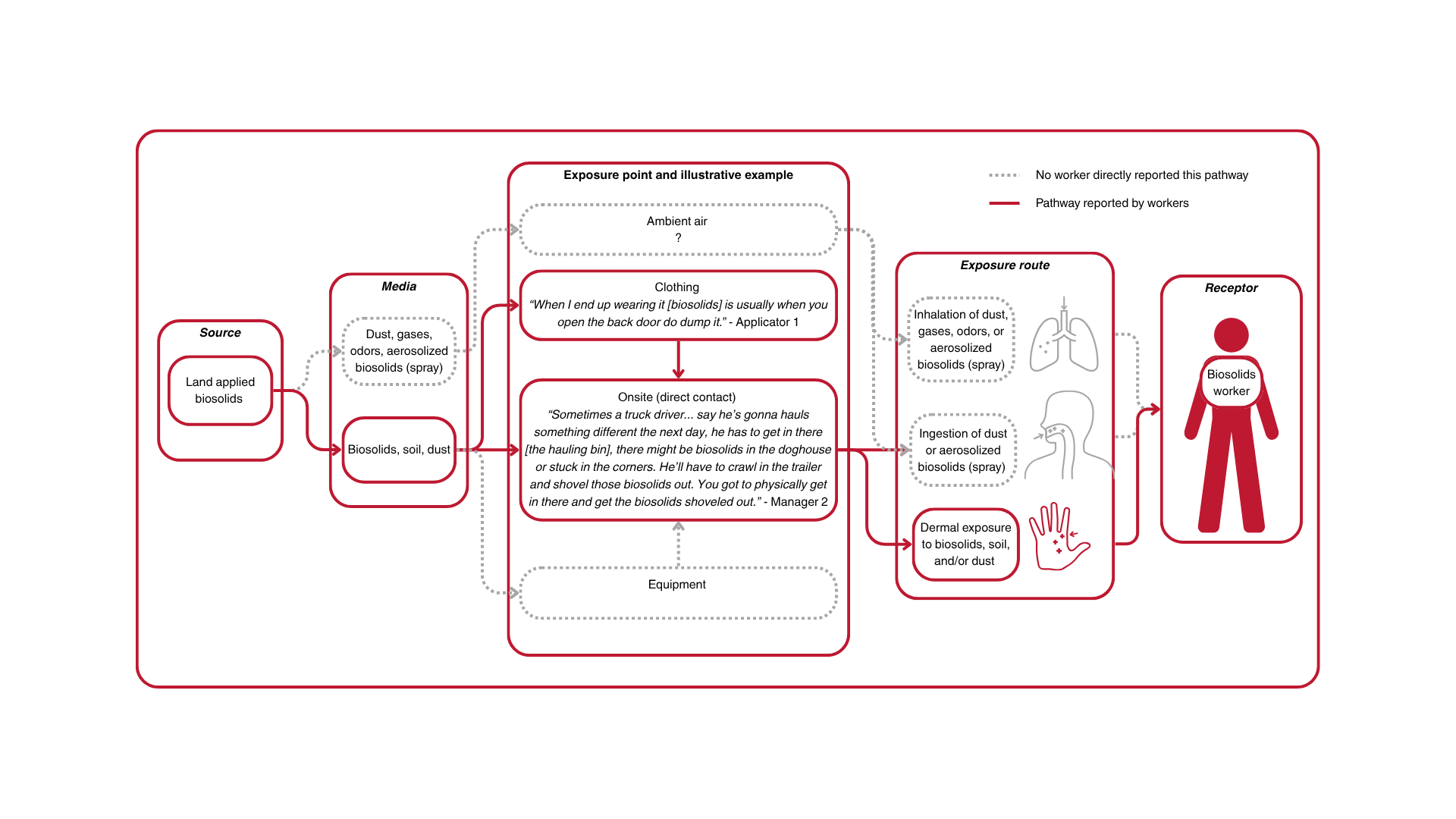


**Figure S2.** Completed exposure pathways relevant to loading tasks


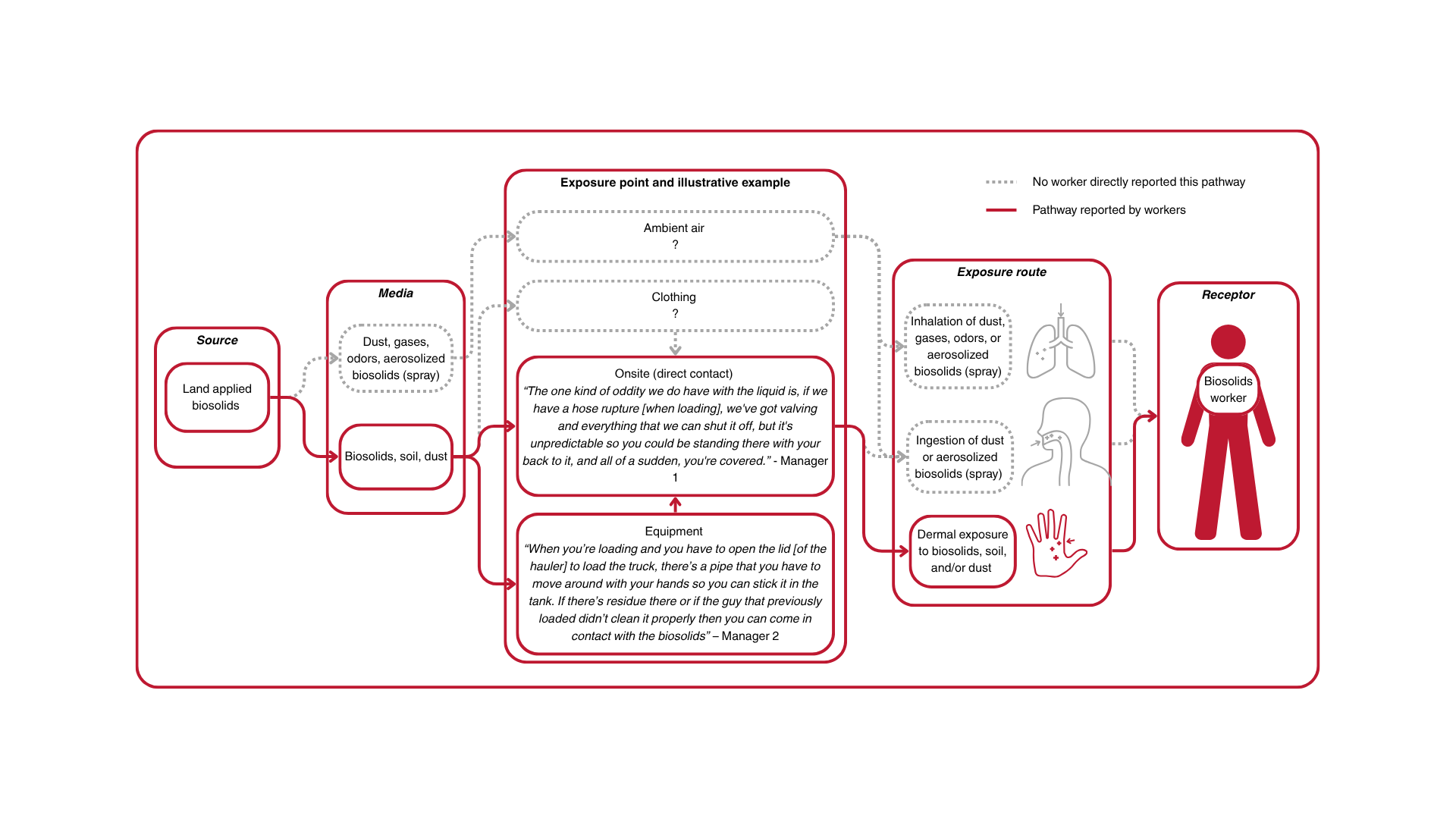


**Figure S3.** Completed exposure pathways relevant to post-application field work tasks


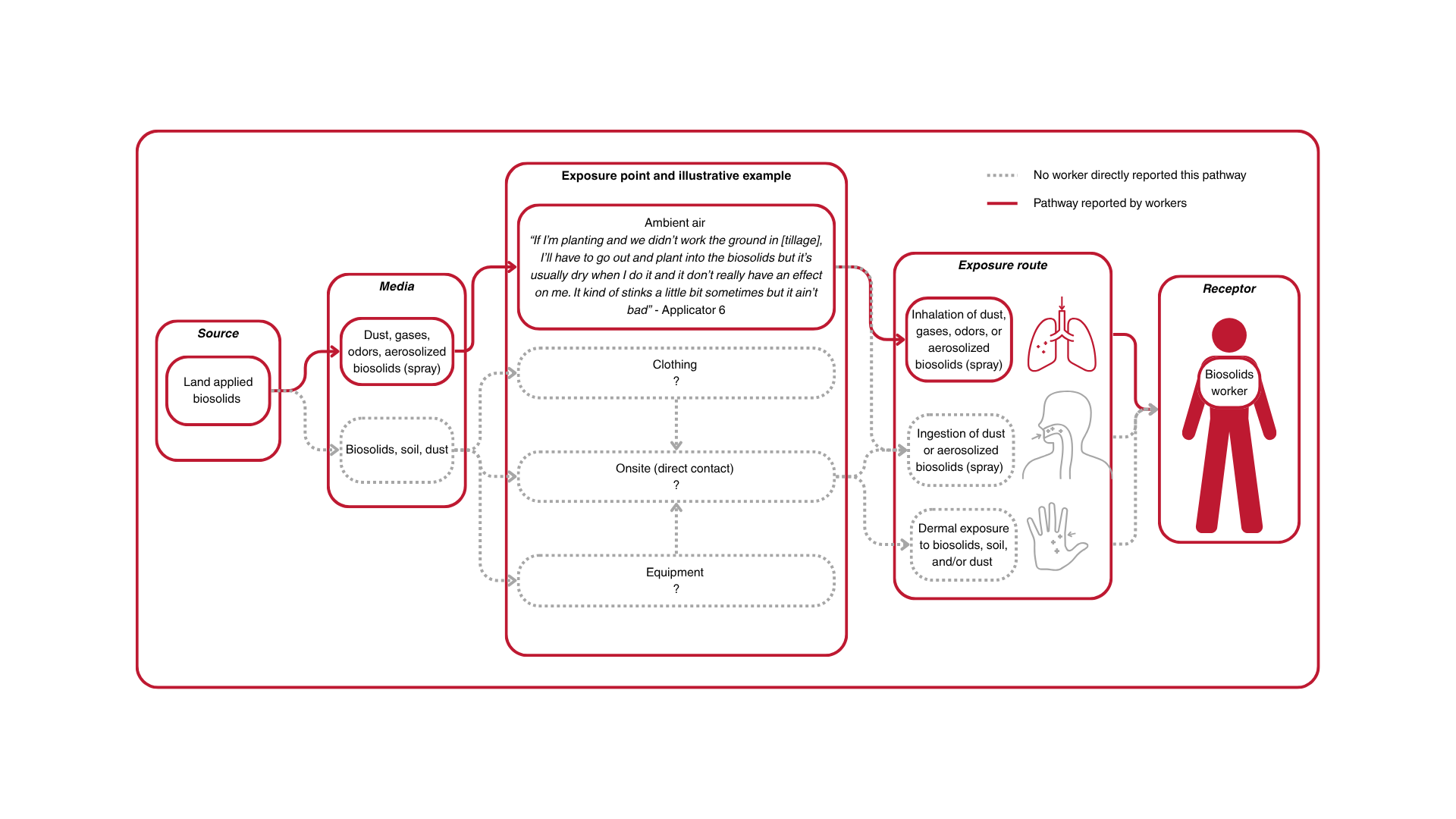


**Figure S4.** Completed exposure pathways relevant to maintenance tasks


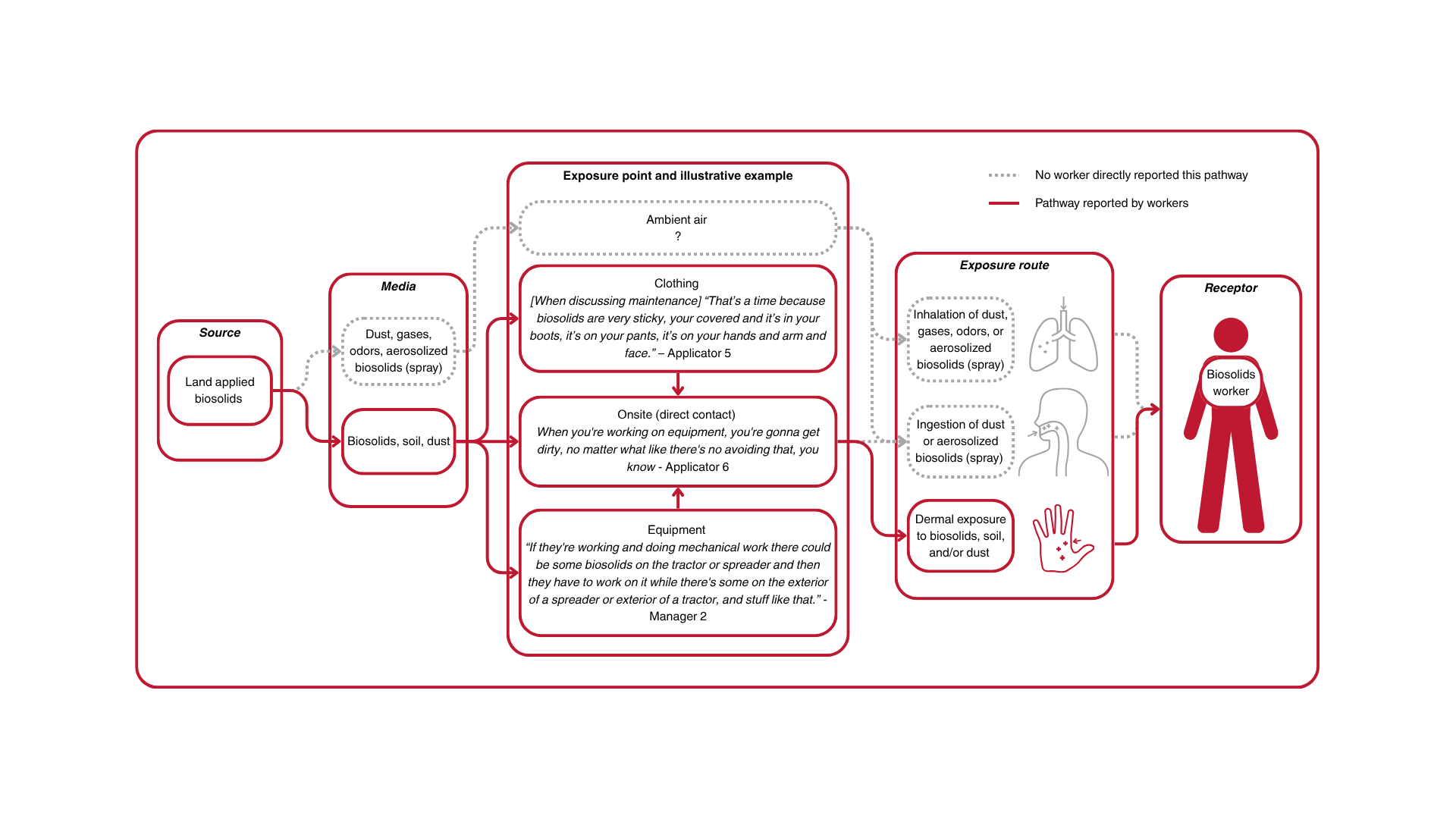


**Supplementary Information**: In-depth interview guide

| **Introduction and work activities**   1. Tell me about your work [probe for information on experience working at current job]    1. Example probes:       1. What lead you to start working with biosolids?       2. Tell me a bit about the different site(s) you go to apply biosolids.       3. Who do you typically work with in a day? How do you divide work among employees? 2. Describe a typical “day in the life” at your job [probe with questions looking at daily tasks and timing of said tasks]    1. Example probes:       1. When you start your day, is there a list of tasks you need to complete? Who sets that schedule? Do you complete all your tasks in a day?       2. What are some tasks that you do every day? [following up] Can you describe the steps involved in task ‘X’ for me? How long does task ‘X’ take?       3. What are some tasks that you seldom do? When do you do ‘X’ task? How often?       4. Do your daily tasks vary between seasons? [following up] What tasks do you do every season? What tasks vary? Can you describe a typical day in the fall, winter, summer, and spring?       5. In a day, how much of your time is spent in the field? [specify to job title] 3. Can you talk about ‘X’? Explain how you do this. (following up with daily tasks) 4. Can you tell me about your work schedule? [probe with frequency of work questions]    1. Example probes:       1. What does a week look like for you during each season [fall, winter, spring, summer]? 5. What are you specifically in charge of in your role? [segway into daily activities]    1. Follow up:       1. You mentioned you oversaw ‘X’. I am unfamiliar with that; can you describe it to me?       2. [for agricultural workers] What crops do you typically work with? Can you describe the process of planting, growing, and harvesting ‘X’ crop? 6. What kind of tools do you work with at your job?    1. Follow up:       1. What do you use ‘X’ for? How often do you work with ‘X’? |
| --- |
| **Biosolids specific tasks**   1. What kind of biosolids application process do you use? (Injection, incorporation, etc.)    1. Follow up:       1. What determines the method of application?       2. Can you tell me about an application event in as much detail as possible.    2. Follow up:       1. Can you elaborate on ‘X’ (repeat for unfamiliar terms/processes)       2. [for agricultural workers] Explain how biosolids are used on your land/farm    3. Follow up:       1. Talk to me about the timeline of application. How does an application event fit in to planting, growing, harvesting, etc. |
| **Understanding biosolids contact**   1. Describe from start to finish how you come into contact with biosolids. 2. Can you tell me a story about a specific event that was outside of normal protocol? 3. During the workday, do you have a breakfast/lunch/dinner break?    1. Follow up:       1. [if yes] where do you typically eat?       2. Do you ever eat on site?       3. Do you wash your hands before eating? 4. Of the tasks we discussed, which one(s) involve the most soil/biosolids amended soil contact? 5. Do you have any specific actions you take to reduce or increase your contact with biosolids while at work?    1. Follow up:       1. How often do you ‘X’?       2. Why do you ‘X’? 6. Does your employer provide a restroom or hand cleaning facilities? 7. Does your workplace have any policies that provide or require specific clothing or personal protective equipment (gloves, masks, etc.) while working?    1. Follow up:       1. Are these guidelines typically followed? Why or why not? 8. Can you talk to me about what you typically wear to work?    1. Follow up:       1. Do you only wear ‘X’ to work?       2. At the end of the day what do you do with your work clothes? |
| **Safety Concerns**   1. What training did you receive prior to or as part of your employment? 2. Do you have any concerns about your safety or health when working?    1. Follow up:       1. [if yes] what is your greatest concern? |
| **Conclusion**  We want to help understand workers' interactions with biosolids and biosolids soils.   1. Is there anything that you think we should know that we haven’t covered yet? 2. Of all the things we discussed, what is the most important thing that you think I should know? |

**Supplementary Information**: Biosolids Exposure Questionnaire

| **Work-Time Questions**   1. What is your job title? [Open ended] 2. How many employers do you have? [Enter answer – numerical value] 3. In the last 30 days, how many days did you work with or in the presence of biosolids or biosolids amended soil? [Enter answer – numerical value] 4. In the last 30 days, how many hours per day did you typically work with or in the presence of biosolids or biosolids amended soil? [Enter answer – numerical value] 5. On the days you worked with biosolids, where did you spend your time? Please check all that apply and estimate the percentage of time you spent in each place. (Check all that apply) (Validation = 100%)    1. Outside (0-100%)    2. Vehicle or cab space (0-100%)    3. Office (0-100%)    4. Other [Open ended] |
| --- |
| **Activity Specific Questions**  *Next, I’m going to ask you about the parts (or tasks) of the process you are directly involved in. For our purposes, we have broken the application process down into six tasks: hauling, loading, spreading, post-application field work, cleaning, and maintenance.*   1. In the last year, what tasks did you perform in the biosolids application process? (Check all that apply)    1. Hauling – Includes picking up biosolids from wastewater treatment facility and delivering biosolids to application site    2. Loading– Loading the spreader with biosolids    3. Spreading– Spreading biosolids on field or application site    4. Post-application field work – Incorporating or tilling the biosolids or planting crops or cover crops    5. Cleaning - Cleaning biosolids off equipment, roads, public space, inside cab space, etc.    6. Maintenance – Performing maintenance or fixing breakages on hauling, loading, or spreading equipment   If [a,b,c,d,e]   1. In the last year, in what month(s) did you [a,b,c,d,e] biosolids? (Check all that apply0    1. January    2. February    3. March    4. April    5. May    6. June    7. July    8. August    9. September    10. October    11. November    12. December   *Now I want to ask more specifically about what you did in just the last 30 days.*  If [a,b,c,d,e]   1. Did you [a,b,c,d,e] biosolids in the last 30 days?    1. Yes    2. No |
| **If hauling (trucking) in the last 30 days = Yes**  *Hauling or trucking begins when you arrive at the wastewater treatment facility or source of biosolids with an empty truck or tanker and ends when you dump or unload the biosolids at the field or site of application.*   1. In the last 30 days, how many days did you haul biosolids [Enter answer – numerical value] 2. On the days that you hauled biosolids, how many hours does it typically take per day? [Enter answer – numerical value] 3. On the days that you hauled biosolids, how long did each of the following actions typically take you (in minutes)?    1. Loading the hauler at the wastewater treatment facility [Enter number (minutes)]    2. Driving the full hauler to the wastewater treatment facility [Enter number (minutes)]    3. Unloading the hauler at the application site [Enter number (minutes)] 4. What equipment do you use to haul the biosolids? (Check all that apply)    1. Dump truck (open top)    2. Semi-truck (enclosed truck)    3. Tanker (liquid)    4. Other [Open ended] 5. What do you wear when you are hauling biosolids? (Check all that apply)    1. Hat or head covering    2. Protective Eyewear    3. Mask (Please specify)       1. What kind of mask do you wear when hauling?          1. Cloth mask          2. Disposable face mask          3. Filtering facepiece respirator (KN95, N95)          4. Elastomeric half or qurter mask respirator (vapor mask)    4. Face shield    5. Long pants    6. Short pants    7. Long sleeve shirt or jacket    8. Short sleeve shirt    9. Sneakers    10. Boots    11. Other [Open ended] 6. In the last 30 days, what percentage of the time do you typically wash your hands immediately after hauling?    1. 0-100% (sliding scale) 7. In the last 30 days, when you hauled biosolids, did you ever notice biosolids... (Check all that apply)    1. In your mouth    2. On your face or head    3. On your hands    4. On another part of your skin or body (unclothed)    5. On your clothes   If in your mouth (a)   1. In the last 30 days, how many times have you noticed biosolids in your mouth while you are hauling? [Enter answer – Numerical value)   If on your face or head, hands, on another part of your body, or on your clothes (b-e)   1. In the last 30 days, how many times have you noticed biosolids _____ (fill in above answer) while you are hauling? [Enter answer – Numerical value) 2. In the last 30 days, what percentage of time spent hauling do you have biosolids _____ (fill in above answer)    1. 0-100% (sliding scale) 3. In the last 30 days, what percentage of the time spent hauling can you smell the biosolids?    1. 0-100% (Sliding Scale) 4. When you are hauling biosolids, how would you characterize your exposure to biosolids on a scale of 1-10, 1 being no contact at all and 10 being high contact.    1. 1-10 (Sliding scale) |
| **If loading in the last 30 days = Yes**  *Loading begins when you start loading the spreader with the biosolids and ends when the spreader leaves the loading site to begin spreading.*   1. In the last 30 days, how many days did you load the spreader? [Enter answer - numerical value] 2. On the days that you loaded biosolids, how many hours does it typically take per day? [Enter answer – numerical value] 3. What equipment do you use to load the spreader (Check all that apply)    1. Hose    2. Vacuum suction    3. Skid loader    4. Backhoe loader    5. Track loader    6. Swing loader    7. Shovel (manual)    8. Other [Open ended] 4. What do you wear when you are loading the spreader (Check all that apply)    1. Hat or head covering    2. Protective Eyewear    3. Mask (Please specify)       1. What kind of mask do you wear when hauling?          1. Cloth mask          2. Disposable face mask          3. Filtering facepiece respirator (KN95, N95)          4. Elastomeric half or qurter mask respirator (vapor mask)    4. Face shield    5. Long pants    6. Short pants    7. Long sleeve shirt or jacket    8. Short sleeve shirt    9. Sneakers    10. Boots    11. Other [Open ended] 5. In the 30 days, what percentage of the time do you typically wash your hands immediately after loading the spreader?    1. 0-100% (sliding scale) 6. In the last 30 days when you loaded the spreader, did you ever notice biosolids... (Check all that apply)    1. In your mouth    2. On your face or head    3. On your hands    4. On another part of your body (Unclothed)    5. On your clothes   If in your mouth (a)   1. In the last 30 days, how many times have you noticed biosolids in your mouth while you are loading [Enter answer – numerical value]   If on your face or head, hands, on another part of your body, or on your clothes (b-e)   1. In the last 30 days, how many times have you noticed biosolids ______ (fill in above answer) while you are loading?   [Enter answer – Numerical value) 2. In the last 30 days, what percentage of time spent loading the spreader do you have biosolids _____ (fill in above answer)    1. 0-100% (sliding scale) 3. In the last 30 days, what percentage of the time spent loading the spreader can you smell the biosolids?    1. 0-100% (Sliding Scale) 4. When you are loading biosolids, how would you characterize your exposure to biosolids on a scale of 1-10, 1 being no contact at all and 10 being high contact.    1. 1-10 (Sliding scale) |
| **If spreading in the last 30 days = Yes**  *Spreading begins when the spreader leaves the loading zone and ends when the spreader is completely emptied on the field.*   1. In the last 30 days, how many days did you spread biosolids? [Enter answer - numerical value] 2. On the days that you spread biosolids, how many hours does it typically take per day? [Enter answer – numerical value] 3. What equipment do you use to spread the biosolids (Check all that apply)    1. Injectors    2. Manure spreaders    3. Vertical beater    4. Horizontal beater    5. Other [Open ended] 4. When you spread biosolids on the field, where do they exit the vehicle in relation to the cab?    1. In front of the cab    2. Behind the cab    3. To the right of the cab    4. To the left of the cab 5. What do you wear when you are spreading (Check all that apply)    1. Hat or head covering    2. Protective Eyewear    3. Mask (Please specify)       1. What kind of mask do you wear when hauling?          1. Cloth mask          2. Disposable face mask          3. Filtering facepiece respirator (KN95, N95)          4. Elastomeric half or quarter mask respirator (vapor mask)    4. Face shield    5. Long pants    6. Short pants    7. Long sleeve shirt or jacket    8. Short sleeve shirt    9. Sneakers    10. Boots    11. Other [Open ended] 6. In the 30 days, what percentage of the time do you typically wash your hands immediately after spreading biosolids?    1. 0-100% (sliding scale) 7. In the last 30 days when you spread biosolids did you ever notice biosolids... (Check all that apply)    1. In your mouth    2. On your face or head    3. On your hands    4. On another part of your body (Unclothed)    5. On your clothes   If in your mouth (a)   1. In the last 30 days, how many times have you noticed biosolids in your mouth while you are spreading biosolids [Enter answer – numerical value]   If on your face or head, hands, on another part of your body, or on your clothes (b-e)   1. In the last 30 days, how many times have you noticed biosolids ______ (fill in above answer) while you are spreading biosolids?   [Enter answer – Numerical value) 2. In the last 30 days, what percentage of time spent spreading do you have biosolids _____ (fill in above answer)    1. 0-100% (sliding scale) 3. In the last 30 days, what percentage of the time spent spreading can you smell the biosolids?    1. 0-100% (Sliding Scale) 4. When you are spreading the biosolids, how would you characterize your exposure to biosolids on a scale of 1-10, 1 being no contact at all and 10 being high contact    1. 1-10 (Sliding scale) |
| **If post-application field work in the last 30 days = Yes**  *Post-application field work includes anything you do to the field after biosolids have been applied. This may include incorporating the biosolids, tilling, or planting of crops or cover crops.*   1. In the last 30 days, how many days did you do post-application field work? [Enter answer - numerical value] 2. On the days that you did post-application field work, how many hours does it typically take per day? [Enter answer – numerical value] 3. What equipment do you use to do post-application field work?    1. [Open ended] 4. What do you wear when you are doing post-application field work (Check all that apply)    1. Hat or head covering    2. Protective Eyewear    3. Mask (Please specify)       1. What kind of mask do you wear when hauling?          1. Cloth mask          2. Disposable face mask          3. Filtering facepiece respirator (KN95, N95)          4. Elastomeric half or quarter mask respirator (vapor mask)    4. Face shield    5. Long pants    6. Short pants    7. Long sleeve shirt or jacket    8. Short sleeve shirt    9. Sneakers    10. Boots    11. Other [Open ended] 5. In the 30 days, what percentage of the time do you typically wash your hands immediately after doing post-application field work?    1. 0-100% (sliding scale) 6. In the last 30 days when you did post-application field work did you ever notice biosolids... (Check all that apply)    1. In your mouth    2. On your face or head    3. On your hands    4. On another part of your body (Unclothed)    5. On your clothes   If in your mouth (a)   1. In the last 30 days, how many times have you noticed biosolids in your mouth while you are doing post-application field work [Enter answer – numerical value]   If on your face or head, hands, on another part of your body, or on your clothes (b-e)   1. In the last 30 days, how many times have you noticed biosolids ______ (fill in above answer) while you are doing post-application field work?   [Enter answer – Numerical value) 2. In the last 30 days, what percentage of time spent doing post-application field work do you have biosolids _____ (fill in above answer)    1. 0-100% (sliding scale) 3. Out of the time you are in the field doing post-application field work, what percentage of the time can you smell the biosolids?    1. 0-100% (Sliding Scale) 4. When you are doing post-application field work, how would you characterize your exposure to biosolids on a scale of 1-10, 1 being no contact at all and 10 being high contact    1. 1-10 (Sliding scale) |
| **If cleaning in the last 30 days = Yes**  *Cleaning includes any time that you must clean biosolids off of equipment, roads, public spaces, the inside of cabs, carriers, etc.*   1. In the last 30 days, how many days did you clean biosolids off equipment, roads, public spaces, the inside of cabs, carriers, etc.?   [Enter answer - numerical value] 2. On the days that you cleaned, how many hours does it typically take per day? [Enter answer – numerical value] 3. What equipment do you typically use to clean? (Check all that apply)    1. Power washer    2. Hose    3. Shovel    4. Rake    5. Stick    6. Other [Open ended] 4. What do you typically wear when you are cleaning? (Check all that apply)    1. Hat or head covering    2. Protective Eyewear    3. Mask (Please specify)       1. What kind of mask do you wear when hauling?          1. Cloth mask          2. Disposable face mask          3. Filtering facepiece respirator (KN95, N95)          4. Elastomeric half or quarter mask respirator (vapor mask)    4. Face shield    5. Long pants    6. Short pants    7. Long sleeve shirt or jacket    8. Short sleeve shirt    9. Sneakers    10. Boots    11. Other [Open ended] 5. In the 30 days, what percentage of the time do you typically wash your hands immediately after cleaning?    1. 0-100% (sliding scale) 6. In the last 30 days when you cleaned, did you ever notice biosolids... (Check all that apply)    1. In your mouth    2. On your face or head    3. On your hands    4. On another part of your body (Unclothed)    5. On your clothes   If in your mouth (a)   1. In the last 30 days, how many times have you noticed biosolids in your mouth while you are cleaning? [Enter answer – numerical value]   If on your face or head, hands, on another part of your body, or on your clothes (b-e)   1. In the last 30 days, how many times have you noticed biosolids ______ (fill in above answer) while you are cleaning?   [Enter answer – Numerical value) 2. In the last 30 days, what percentage of time spent cleaning do you have biosolids _____ (fill in above answer)    1. 0-100% (sliding scale) 3. In the last 30 days, what percentage of the time spent cleaning can you smell the biosolids?    1. 0-100% (Sliding Scale) 4. When you are spreading the biosolids, how would you characterize your exposure to biosolids on a scale of 1-10, 1 being no contact at all and 10 being high contact    1. 1-10 (Sliding scale) |
| **If maintenance in the last 30 days = Yes**  *Maintenance includes any time that you perform routine maintenance or repair biosolids equipment including spreaders, tankers, haulers, loaders, or other pieces of equipment involved in the application process.*   1. In the last 30 days, how many days did you perform routine maintenance or repair biosolids equipment? [Enter answer - numerical value] 2. On the days that you did maintenance, how many hours does it typically take per day? [Enter answer – numerical value] 3. What do you typically wear when you are doing maintenance? (Check all that apply)    1. Hat or head covering    2. Protective Eyewear    3. Mask (Please specify)       1. What kind of mask do you wear when hauling?          1. Cloth mask          2. Disposable face mask          3. Filtering facepiece respirator (KN95, N95)          4. Elastomeric half or quarter mask respirator (vapor mask)    4. Face shield    5. Long pants    6. Short pants    7. Long sleeve shirt or jacket    8. Short sleeve shirt    9. Sneakers    10. Boots    11. Other [Open ended] 4. In the 30 days, what percentage of the time do you typically wash your hands immediately after doing maintenance?    1. 0-100% (sliding scale) 5. In the last 30 days when you performed routine maintenance or repaired equipment, did you ever notice biosolids... (Check all that apply)    1. In your mouth    2. On your face or head    3. On your hands    4. On another part of your body (Unclothed)    5. On your clothes   If in your mouth (a)   1. In the last 30 days, how many times have you noticed biosolids in your mouth while you are doing maintenance? [Enter answer – numerical value]   If on your face or head, hands, on another part of your body, or on your clothes (b-e)   1. In the last 30 days, how many times have you noticed biosolids ______ (fill in above answer) while you are doing maintenance?   [Enter answer – Numerical value) 2. In the last 30 days, what percentage of time spent doing maintenance do you have biosolids _____ (fill in above answer)    1. 0-100% (sliding scale) 3. In the last 30 days, what percentage of the time spent doing maintenance can you smell the biosolids?    1. 0-100% (Sliding Scale) 4. When you are doing maintenance, how would you characterize your exposure to biosolids on a scale of 1-10, 1 being no contact at all and 10 being high contact    1. 1-10 (Sliding scale) |
| **Class, Type, and Form of Biosolids**   1. What class of biosolids do you work with? (Check all that apply)    1. Class A    2. Class B    3. Exceptional Quality (EQ)    4. Other [Open ended]    5. I don’t know   If multiple, specify the percentage of each class that you work with (Validation = 100%)   - 1. Class A (0-100)   2. Class B (0-100)   3. Exceptional Quality (0-100)   4. Other (0-100)  1. What type of biosolids do you work with (Check all that apply)    1. Digested biosolids    2. Lime amended biosolids    3. Composted biosolids    4. I don’t know    5. Other [Open ended] 2. What is the consistency of the biosolids you work with (Check all that apply)    1. Liquid (<3% solids)    2. Semi-liquid (3-15% solids)    3. Cake like (15-30% solids)    4. Dry, sticky (30-50% solids)    5. Dry, pellets (50-100% solids)   If liquid   1. When you are working with liquid biosolids, how would you characterize your exposure to biosolids on a scale of 1-10, 1 being no contact at all and 10 being high contact.    1. 1-10 (Sliding scale)   If semi-liquid   1. When you are working with semi-liquid biosolids, how would you characterize your exposure to biosolids on a scale of 1-10, 1 being no contact at all and 10 being high contact.    1. 1-10 (Sliding scale)   If cake like   1. When you are working with cake like biosolids, how would you characterize your exposure to biosolids on a scale of 1-10, 1 being no contact at all and 10 being high contact.    1. 1-10 (Sliding scale)   If dry, sticky   1. When you are working with dry, sticky biosolids, how would you characterize your exposure to biosolids on a scale of 1-10, 1 being no contact at all and 10 being high contact.    1. 1-10 (Sliding scale)   If dry, pellets   1. When you are working with dry, pellet biosolids, how would you characterize your exposure to biosolids on a scale of 1-10, 1 being no contact at all and 10 being high contact.    1. 1-10 (Sliding scale) 2. After a day working with or in the presence of biosolids, what do you typically do with your work clothes [Open ended] |
| **Other activities**   1. We are interested in knowing more about the processes involved in land application and where you are involved. Earlier, we broke this down into hauling, loading, spreading, post-application field work, cleaning, and maintenance.  Is there anything you typically do during a normal day that we haven’t covered yet?    1. Yes    2. No    3. I don’t know   If yes   1. Please specify the activity(s) we didn’t cover [Open ended] 2. When you do this (referring to above), how would you characterize your exposure to biosolids on a scale of 1-10, 1 being no contact at all and 10 being high contact.    1. 1-10 (Sliding scale) |
| **Typicalness Questions**   1. On a scale of 1(completely atypical) - 5(very typical), how typical was this past year for you?    1. 1: Not typical    2. 2: Mostly typical    3. 3: Somewhat typical    4. 4: Typical    5. 5: Very typical   If 3 or less,   1. What made this year less “typical” than normal [Open ended] 2. On a scale of 1(completely atypical) - 5(very typical), how typical was the last 30 days for you?    1. 1: Not typical    2. 2: Mostly typical    3. 3: Somewhat typical    4. 4: Typical    5. 5: Very typical   If 3 or less,   1. What made the last 30 days less “typical” than normal [Open ended] |

**Supplementary Information**: Demographic Questionnaire

| 1. What year were you born [Enter answer – numerical value] 2. What is your assigned sex at birth    1. Male    2. Female    3. Prefer not to state 3. How do you identify?    1. Male    2. Female    3. Non-binary    4. Genderqueer    5. Other [Open ended]    6. Prefer not to state 4. What is your highest attained education level?    1. Less than high school completion    2. High school completion    3. Some college    4. Associates degree    5. Bachelor’s degree    6. Master’s degree    7. Doctoral degree 5. What state do you work in (enter multiple, if necessary) [Open ended] 6. What is your official job title? [Open ended] 7. How long have you worked with biosolids? [Enter answer – numerical value] 8. How long have you worked in your current role? [Enter answer – numerical value] |
| --- |
